# The Alberta moving beyond breast cancer (AMBER) cohort study: baseline description of the full cohort

**DOI:** 10.1007/s10552-021-01539-6

**Published:** 2022-01-22

**Authors:** Christine M. Friedenreich, Jeff K. Vallance, Margaret L. McNeely, S. Nicole Culos-Reed, Charles E. Matthews, Gordon J. Bell, John R. Mackey, Karen A. Kopciuk, Leanne Dickau, Qinggang Wang, Diane Cook, Stephanie Wharton, Jessica McNeil, Charlotte Ryder-Burbidge, Andria R. Morielli, Kerry S. Courneya

**Affiliations:** 1 Department of Cancer Epidemiology and Prevention Research, Cancer Care Alberta, Alberta Health Services, Holy Cross Center, 2210-2nd St SW, Calgary, AB T2S 3C3 Canada; 2 Department of Oncology, Cumming School of Medicine, University of Calgary, Calgary, AB Canada; 3 Present Address: Department of Community Health Sciences, Cumming School of Medicine, University of Calgary, Calgary, AB Canada; 4 Faculty of Health Disciplines, Athabasca University, Athabasca, AB Canada; 5 Faculty of Rehabilitation Medicine, University of Alberta, Edmonton, AB Canada; 6 Faculty of Kinesiology, University of Calgary, Calgary, AB Canada; 7 Division of Cancer Epidemiology and Genetics, US National Cancer Institute, Rockville, MD USA; 8 Faculty of Kinesiology, Sport, and Recreation, College of Health Sciences, University of Alberta, Edmonton, AB Canada; 9 Faculty of Medicine and Dentistry, University of Alberta, Edmonton, AB Canada; 10 Department of Mathematics and Statistics, Faculty of Science, University of Calgary, Calgary, AB Canada; 11 Department of Kinesiology, School of Health and Human Sciences, University of North Carolina at Greensboro, Greensboro, NC USA

**Keywords:** Breast cancer, Physical activity, Sedentary behavior, Fitness, Survival, Cohort study

## Abstract

**Purpose:**

The Alberta Moving Beyond Breast Cancer (AMBER) Study is an ongoing prospective cohort study investigating how direct measures of physical activity (PA), sedentary behavior (SB), and health-related fitness (HRF) are associated with survival after breast cancer.

**Methods:**

Women in Alberta with newly diagnosed stage I (≥ T1c) to IIIc breast cancer were recruited between 2012 and 2019. Baseline assessments were completed within 90 days of surgery. Measurements included accelerometers to measure PA and SB; a graded treadmill test with gas exchange analysis to measure cardiorespiratory fitness (VO_2peak_); upper and lower body muscular strength and endurance; dual-X-ray absorptiometry to measure body composition; and questionnaires to measure self-reported PA and SB.

**Results:**

At baseline, the 1528 participants’ mean age was 56 ± 11 years, 59% were post-menopausal, 62% had overweight/obesity, and 55% were diagnosed with stage II or III disease. Based on device measurements, study participants spent 8.9 ± 1.7 h/day sedentary, 4.4 ± 1.2 h/day in light-intensity activity, 0.9 ± 0.5 h/day in moderate-intensity activity, and 0.2 ± 0.2 h/day in vigorous-intensity activity. For those participants who reached VO_2peak_, the average aerobic fitness level was 26.6 ± 6 ml/kg/min. Average body fat was 43 ± 7.1%.

**Conclusion:**

We have established a unique cohort of breast cancer survivors with a wealth of data on PA, SB, and HRF obtained through both direct and self-reported measurements. Study participants are being followed for at least ten years to assess all outcomes after breast cancer. These data will inform clinical and public health guidelines on PA, SB, and HRF for improving breast cancer outcomes.

**Supplementary Information:**

The online version contains supplementary material available at 10.1007/s10552-021-01539-6.

## Introduction

In 2020, approximately 27,700 Canadian women were diagnosed with breast cancer and 5,100 died from the disease [1]. While incidence rates have remained stable over recent decades, mortality rates have improved since their peak in the mid-1980s and are now projected to be 22 per 100,000 for Canadian women. Decreases in mortality rates can be attributed to improved early detection and treatments. The current five-year relative survival rate after breast cancer is 88% with an estimated 400,000 breast cancer survivors currently alive in Canada [2].

Despite improved survival rates, life after breast cancer remains challenging given the multimodal therapy that is sometimes difficult and prolonged, resulting in negative effects on the long-term health and well-being of breast cancer survivors. After breast cancer treatments, survivors face increased risks of recurrence, second cancers, cardiac dysfunction, weight gain, bone loss, lymphedema, arthralgias, cognitive dysfunction, menopausal symptoms, fatigue, and psychosocial distress [3–7].

Observational epidemiologic studies have shown that self-reported physical activity either before or after a breast cancer diagnosis is associated with improved survival outcomes [8]. A recent meta-analysis of physical activity and cancer survival suggested the highest levels of post-diagnosis physical activity (compared to the lowest levels) were associated with a hazard ratio of 0.63 (95% CI = 0.50–0.78) for breast cancer-specific mortality and 0.58 (95% CI = 0.52–0.65) for all-cause mortality [8]. In general population cohorts, higher levels of sedentary behavior (time spent sitting, lying, or standing with energy expenditure below 1.5 metabolic equivalents of task (METs)) have been associated with increased cancer mortality rates [9] and there is evidence that physical activity may modify these associations [10]. Compared to individuals without cancer, breast cancer survivors have significantly higher odds of spending ≥ 8 h per day engaged in self-report sedentary behavior (OR = 1.99; 95% CI = 1.25–3.19) [11]. Contrarily, breast cancer survivors have similar levels of accelerometer-assessed sedentary behavior compared with non-cancer survivors [12]. Limited research has examined associations between sedentary behavior and outcomes in cancer survivors. A recent meta-analysis identified 29 studies, nine of which included mortality outcomes [13]. Compared to the lowest level of post-diagnosis sedentary behavior, the highest level of post-diagnosis sedentary behavior was associated with a hazard ratio of 1.22 (95% CI = 1.06–1.41) for all-cause mortality.

The primary health-related fitness parameter (i.e., body composition, cardiopulmonary fitness, muscular strength and endurance, flexibility, and balance) that has been examined for its association with breast cancer survival is body mass index (BMI, kg/m^2^). A systematic review [14] of BMI and mortality in breast cancer included 79 studies that assessed BMI within 12 months of diagnosis. Breast cancer-specific and all-cause mortality were increased for women with a BMI ≥ 30 (compared to those with a BMI < 30) with relative risks of 1.25 (95% CI = 1.10–1.42) and 1.23 (95% CI = 1.12–1.33). This review found that higher BMI assessed at ≥ 12 months after diagnosis was associated with greater breast cancer mortality risk than the risk associated with obesity closer to diagnosis. These results highlight the importance of considering the association of other health-related fitness parameters on breast cancer outcomes over the cancer continuum.

Most observational studies have captured physical activity by self-report and included only limited measurements of sedentary behavior and health-related fitness. These studies have not measured changes in exposures over time. We previously described our Alberta Moving Beyond Breast Cancer (AMBER) Study cohort that was specifically designed to address these gaps in knowledge regarding the role of physical activity, sedentary behavior, and health-related fitness in breast cancer survivors from diagnosis to end-of-life [15]. We also have reported on the feasibility of recruitment into this prospective cohort with the first 500 participants [16]. The aim of this paper is to report the baseline descriptive data for the primary exposures of interest in the full cohort of 1,528 AMBER study participants.

## Methods

### Study design and participant recruitment

A description of the AMBER study design and methods has previously been described [15, 16]. We enrolled the first participant in July 2012 and completed enrollment in July 2019. Women living in Edmonton or Calgary (or surrounding areas), Alberta, Canada with newly diagnosed breast cancer were eligible if they had histologically confirmed stage I (≥ T1c) to stage IIIc breast cancer, were 18 to 80 years old, could complete the revised Physical Activity Readiness Questionnaire for Everyone (rPAR-Q +) [17], were able to complete questionnaires in English, and were not pregnant at the time of recruitment. Potential participants in Calgary were identified through the Alberta Cancer Research Biobank (ACRB) who approached all breast cancer patients at the time of diagnosis and requested a blood sample for the biobank. These women were contacted for the AMBER cohort study once their clinical and pathology results were available to confirm eligibility. In Edmonton, eligible participants were identified through the Cross Cancer Institute’s New Patient Breast Cancer clinics and approached by their treating oncologist at their first visit and introduced to the study. Those who expressed interest were then further screened for eligibility. In both centers, AMBER recruiters explained the study and provided potential participants with study information and followed up via telephone with eligible participants to confirm their interest in the study. Signed written consent was obtained and the rPAR-Q + completed during the first day of testing. Ethics approval was obtained through the Health Research Ethics Board of Alberta: Cancer Committee.

### Timing of assessments and measurements

The AMBER study includes measurements at four time points: baseline, 1-, 3-, and 5-year post-diagnosis. The first three sets of assessments included questionnaires, blood collection, one or two days of in-person health-related fitness testing, and week-long device-based measures of physical activity and sedentary behavior. Only questionnaires are being completed at the 5-year follow-up assessment. The goal was to have participants complete baseline assessments within 90 days of surgery and/or prior to initiating adjuvant systemic or local treatments. Due to personal circumstances, some participants were allowed into the cohort if they had completed up to two cycles of chemotherapy or ten fractions of radiation therapy. Using this approach, we were able to accommodate more participants after determining that women were still willing and able to complete baseline assessments after the start of their treatments. In a subset of women who received neoadjuvant treatment, the goal was to complete baseline assessments prior to initiating chemotherapy but always before the third cycle of chemotherapy.

Four sets of questionnaires were completed at baseline. The *Baseline Health Questionnaire* asked participants about their sociodemographic characteristics, menstrual, reproductive, and medical history, exogenous hormone and medication use history, family history of cancer, and lifetime smoking and alcohol use histories. In addition, the *Canadian adaptation of the US National Cancer Institute’s past year Diet History Questionnaire II (CDHQ-II)* [18], the *Past Year Total Physical Activity Questionnaire (PYTPAQ)* [19], and a *General Health Questionnaire* that measured patient-reported outcomes including health-related quality of life, symptoms (e.g., fatigue), psychosocial outcomes (e.g., anxiety, depression), facilitators and barriers to physical activity, and sedentary behavior were completed [20–29]. Except for the *Baseline Health Questionnaire*, all other questionnaires are being completed at each follow-up time point.

Physical activity and sedentary behavior were further assessed at baseline with 1- and 3-year follow-ups using the waist-worn ActiGraph GT3X + ® (ActiGraph, LLC, Pensacola, FL) and the thigh-worn activPAL® inclinometer (PAL Technologies, Glasgow, Scotland). Participants wore these devices for seven consecutive days and completed an Activity Monitor Log to record when the devices were worn (and removed) each day.

Health-related fitness assessments were performed by certified exercise physiologists using standardized testing protocols and the same equipment at both sites have been previously described [15]. The assessments include resting blood pressure and heart rate; body composition (dual x-ray absorptiometry (DXA), body mass, height, waist and hip circumferences); abdominal endurance (curl-ups); sit and reach flexibility; balance; combined right and left grip strength; cardiorespiratory fitness (graded treadmill exercise test combined with metabolic measurements that included submaximal heart rate, blood pressure, ratings of perceived exertion, ventilatory threshold, VO_2peak_, and recovery heart rate); and upper and lower body muscular strength (chest and leg press predicted one repetition maximum; predicted 1-RM) and endurance (multiple repetition maximum, mRM, based on 50% of predicted 1-RM for the chest press and 70% of predicted 1-RM for the leg press).

Upper body functioning and lymphedema were assessed by self-report in the baseline questionnaire and by direct measurements performed by exercise physiologists. *Arm volume* was assessed using the Perometer® (Perosystems, Germany). Cancer-related lymphedema was considered present with a ≥ 200 ml volume difference between the affected and unaffected arms [30]. Measurements for *shoulder range of motion* (including active and passive measurements of shoulder flexion, abduction, internal rotation, external rotation, and horizontal abduction) indicate a limitation if the difference in range of motion between the affected and unaffected arm exceeds 10°. Self-reported *arm function* was assessed using the Disabilities of the Arm, Shoulder and Hand scale (DASH) [31]. Upper and lower extremity peripheral neuropathy are assessed by self-report and objective measures of sensorimotor function, strength, and balance.

### Blood collection

AMBER study participants were asked to provide a 60-ml fasted blood sample at baseline and 30-ml fasted blood samples at 1- and 3-year follow-up. A standardized protocol for blood collection, processing, shipping, and storage is followed for the collections at both sites. We have 20 aliquots per person per blood draw (4 serum, 12 plasma, 4 buffy coat) available for assays. The aliquoted blood samples are stored in ultralow (− 86 °C) freezers in the ACRB facility in Calgary. Blood collections were done predominantly pre-surgery in Calgary through the ACRB and postsurgically in Edmonton given the timing of recruitment there.

### Data processing and analysis

All self-reported questionnaire data were captured in either TELE*form®* or Blaise® with data verification, cleaning, and analysis done in STATA (version 16) or SAS (version 9.4). Databases in Access® are used for tracking the cohort and for the health-related fitness assessments. Caloric and nutrient intakes from the CDHQ-II are estimated using Diet*Calc [32]. MET values are assigned to each self-reported activity in the PYTPAQ using the Compendium of Physical Activities [33] to derive MET-hours/week for each activity domain (i.e., occupational, household, recreational activities). Sedentary time was estimated as the sum of sitting time (hours/day) for work, travel, and leisure (television, computer, other) both on weekdays and weekends. To capture valid accelerometer wear time, the time spent in bed/sleeping was removed based on the data from the activity monitor logs completed by the participants. The Choi algorithm was used to capture any non-wear time during awake time [34]. Sedentary time, light, moderate, and vigorous-intensity physical activity time were estimated using vertical axis activity counts/minute cut-points and a machine learning technique, combined with a decision tree and artificial neural network (R Sojourn package, Soj3x) [35]. For the activPAL®, sedentary time (sitting/reclining), standing, stepping time, and daily step counts (steps/d) were calculated using activPAL® algorithms (PAL Software version 8). For both devices days with < 10 h of wear time while out of bed for the day was excluded which resulted in four participants’ data being excluded for the activPAL® data only.

Descriptive analyses (means, standard deviation and frequencies, percent) were estimated for all baseline questionnaires, health-related fitness, as well as device-based and self-reported measures of physical activity and sedentary behavior data. Data from the questionnaires were used to further categorize participants into clinically meaningful groups where possible. Menopausal status was determined based on age and a series of questions about the menstrual cycle. Participants were considered post-menopausal at baseline if (1) their period stopped naturally or due to medical treatment, such as surgery, hormone replacement therapy, radiation, or chemotherapy or (2) if they were taking hormone replacement therapy and were > 55 years of age. The burden of co-morbid conditions was estimated using an adapted version of the Charlson Comorbidity Index [36]. Individual conditions were assigned a score of 0.25–6 based on the risk of resource use or mortality and summed to create a single co-morbidity score for each participant.

## Results

**Fig. 1:**
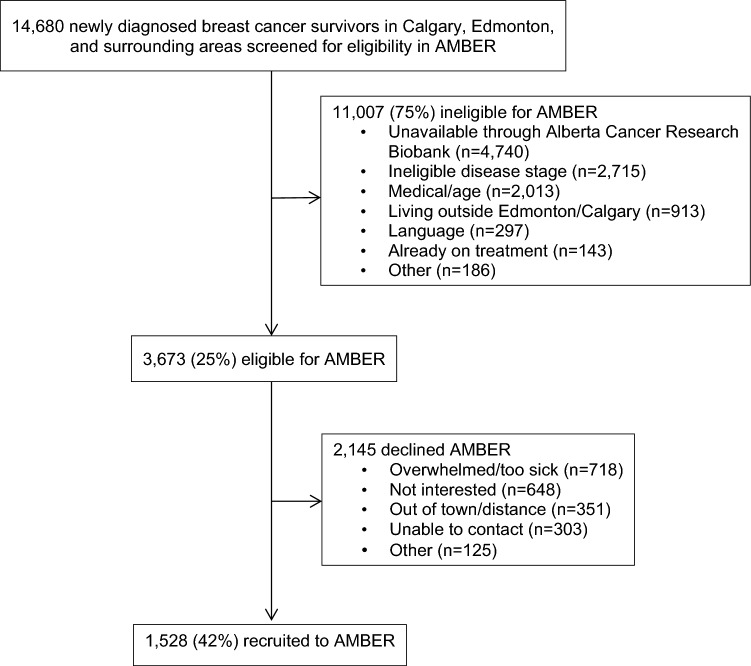
Study flowchart of the 1,528 AMBER Study participants who completed baseline assessments, Alberta, 2012–2019

Between July 2012 and July 2019, we screened 14,680 women with newly diagnosed breast cancer for eligibility and 11,007 were ineligible (Fig. 1). The reasons for ineligibility were not consenting to be contacted for any research after initial contact made by the ACRB (*n* = 4,740; 43%), having an ineligible disease stage (*n* = 2,715; 25%), medical/age issues (e.g., recent stroke, dementia, heart failure, confined to a wheelchair) (*n* = 2,013; 18%), living outside the catchment areas in Calgary and Edmonton (> 200 km from centers) (*n* = 913, 8%), having a language barrier (*n* = 297, 3%), already completed too much adjuvant treatment (*n* = 143, 1%), or other issues (*n* = 186, 1%). We recruited 1,528 of 3,673 eligible (42%) into the study. The most common reasons for refusal were being overwhelmed/too sick (*n* = 718; 20%), not interested (*n* = 648; 18%), and living out of town/distance (*n* = 352; 16%). From the sample, 884 were recruited in Calgary and 644 in Edmonton (Table 1).

**Table 1:** Demographic characteristics of the AMBER cohort at baseline (*N* = 1,528) for the Calgary (*n* = 884) and Edmonton (*n* = 644) sites

Demographic characteristic	Total cohort		Calgary		Edmonton	
	*N* = 1528		*n* = 884		*n* = 644	
	*n*	%	*n*	%	*n*	%
Age at diagnosis, mean, SD	54.9	10.8	55.3	11.3	54.4	10.2
< 40	129	8.4	75	8.5	54	8.4
40–54	610	39.9	350	39.6	260	40.4
55–65	503	32.9	269	30.4	234	36.3
> 65	286	18.7	190	21.5	96	14.9
Race/Ethnicity						
White	1304	87	751	87.1	553	86.8
Asian	104	6.9	61	7.1	43	6.8
Black	11	0.8	5	0.6	7	1.1
Indian	34	2.2	18	2.1	15	2.4
Latin American or Hispanic	18	1.2	14	1.6	4	0.6
First Nations	13	0.9	4	0.5	9	1.4
Don’t know	15	1	9	1	6	0.9
Marital status						
Married or common-law	1131	75.1	641	74.6	490	77.3
Divorced, separated, widowed	272	18.1	164	18.8	108	17
Single (never married)	102	6.8	66	7.6	36	5.7
Highest level of education						
High school or less	335	22.5	185	21.7	150	23.7
College or trade school	472	31.8	264	31	208	32.8
University undergraduate degree/nursing school	390	26.2	233	27.3	157	24.8
University graduate degree	289	19.4	170	20	119	18.8
Income						
≤ $75,000	441	29.2	270	30.8	171	27.1
> $75,000 to ≤ $150,000	538	35.7	285	32.6	253	40.1
> $150,000	403	26.7	237	27.1	166	26.2
Did not report	124	8.2	82	9.4	42	6.6
Menopausal status						
Pre-menopausal	631	41.3	356	40.27	275	42.7
Post-menopausal	897	58.7	528	59.73	369	57.3
Parity, mean, SD	1.7	1.2	1.6	1.3	1.8	1.2
Gravidity, mean, SD	2.3	1.5	2.3	1.5	2.3	1.5
First degree relative with cancer	449	29.4	279	31.6	170	26.4

*SD* standard deviation

Supplementary Table 1 provides details on the baseline assessment completion rates overall and by center for all data collected from the AMBER participants. In total, 1,388 (90.8%) participants attempted the treadmill test and 1,287 (84.2%) completed the test sufficiently to obtain a direct (76.2%) or estimated (8.0%) measure of VO_2peak_. The main reasons for not attempting the treadmill test for 141 participants were because of safety issues, such as the presence of cardiovascular disease risk factors or other health conditions (*n* = 94, 67.1%), illness or musculoskeletal problems, such as knee pain or ankle injury (*n* = 18; 12.9%), or complications related to treatment or surgery (*n* = 14; 10%). The remaining HRF tests were completed by at least 83% of participants with tests having a > 90% completion rate. The HRF assessment completion rates were similar between the two centers except for upper body strength and endurance measures that were completed at a lower rate in Calgary because these participants were recruited into the cohort closer to the time of surgery compared to Edmonton. Baseline HRF assessments were completed after initiation of chemotherapy in 274 (18%) of participants of which 250 had completed one cycle and 24 two cycles of treatment. Likewise, 82 (5%) study participants completed their baseline assessments after starting their radiation treatment with a maximum of 10 fractions.

The baseline assessment completion rates for lymphedema, upper arm function, peripheral neuropathy, and blood collection were over 98% and comparable between centers. Blood samples were obtained from 98.9% of participants with predominantly pre-surgical bloods in Calgary (81.9%) and post-surgical bloods in Edmonton (87.6%) because of the difference in the timing of recruitment between centers. All participants completed the *Baseline Health Questionnaire* and the remaining questionnaires were completed by 95% of participants.

Useable data, defined as ≥ 10 h of wear time/day, were obtained from 95.4% and 94.3% of participants for the ActiGraph GT3X + ® (95.4%) and activPAL® devices.

On average, the AMBER study participants were 54.9 years old (SD = 10.8), predominantly White (87%), married or common-law (75%), well educated (44% with university undergraduate degree or higher), had relatively high family incomes (27% above C$150,000), an average of 1.7 live births (SD = 1.2), were mainly post-menopausal (58.7%), and nearly a third (29%) had a first-degree family history of cancer (Table 1). These characteristics were similar between the two centers.

The AMBER participants’ breast cancer was detected primarily through a diagnostic mammogram (56.1%) (Table 2). The cancer stage at diagnosis was distributed evenly between stage I (44.6%) and stage II (46.6%) with 8.9% of the cohort diagnosed with stage III cancer. Tumor grade was mainly grade 2 (42.9%) and grade 3 (44.5%). AMBER participants were predominantly estrogen receptor positive (88.3%), progesterone receptor positive (76.8%), and 15.6% were HER-2 receptor positive. All but one participant had breast cancer surgery (99.9%), 58.2% were scheduled to receive chemotherapy, 74.2% radiation therapy, 81.6% hormone therapy, and 16% biological therapy. We also had 117 (7.7%) women who were scheduled to receive neoadjuvant treatment. Besides having breast cancer, this cohort had few other co-morbid conditions with less than one other condition reported per participant. These medical and treatment characteristics were similar between centers. The only (small) difference was the higher percentages for chemotherapy, radiation therapy, and neoadjuvant treatments in Edmonton versus Calgary.

**Table 2:** Medical characteristics of the AMBER cohort at baseline (*N* = 1,528) for the Calgary (*n* = 884) and Edmonton (*n* = 644) sites

Medical characteristic	Total cohort		Calgary		Edmonton	
	*N* = 1528		*n* = 884		*n* = 644	
	*n*	%	*n*	%	*n*	%
Charlson Comorbidity Index^a^, median, Q1, Q3	0.5	0, 1.5	0.5	0, 1.5	0.5	0, 1.3
Breast cancer diagnosis						
Method of breast cancer detection						
Routine mammogram	668	43.9	398	45	270	42.3
Diagnostic mammogram	855	56.1	486	55	369	57.8
Breast cancer stage						
Stage I^b^	681	44.6	391	44.3	290	45.0
Stage II	712	46.6	431	48.8	281	43.6
Stage III	134	8.8	61	6.9	73	11.3
Tumor grade^c^						
Grade 1	192	12.6	95	10.8	97	15.0
Grade 2	655	42.9	408	46.2	247	38.3
Grade 3	680	44.5	379	42.9	301	46.7
Estrogen receptor positive	1349	88.3	789	89.2	560	87.0
Progesterone receptor positive	1173	76.8	698	78.9	475	73.8
HER-2 receptor positive	239	15.6	147	16.6	92	14.3
Breast cancer treatment as planned						
Surgery	1527	99.9	883	99.9	644	100
Chemotherapy	889	58.2	486	55.0	403	62.6
Biological therapy	245	16.0	157	17.8	88	13.7
Hormone therapy	1247	81.6	725	82.0	522	81.1
Radiation therapy	1134	74.2	626	70.8	508	78.9
Neoadjuvant treatment	117	7.7	49	5.5	68	10.6

*SD* standard deviation; g, grams, *HER-2* human epidermal growth factor receptor 2

^a^Adapted Charlson Comorbidity Index, where each condition is given a score of 0.25 to 6 and a higher total score represents a higher risk of death or resource use within one year

^b^One participant’s breast cancer stage was downgraded to stage 0 after baseline assessments had been completed

^c^One participant’s breast cancer grade was unknown

Most AMBER participants never smoked (56.7%) with 36.8% reporting being ever smokers (defined as smoking at least 100 cigarettes at some point in their lives) and 6.4% were current smokers (Table 3). This level of smoking was equivalent to 11.2 pack-years of smoking for those who were either current or ever smokers. They also reported an average of 7.1 g of ethanol consumed per day over their lifetimes which is equivalent to about 0.5 drinks of alcohol per day. From the diet history questionnaire, an estimated 1,718 kcals were consumed per day during the past 12 months with daily averages of 72 g of protein, 68 g of fat, and 203 g of carbohydrates.

**Table 3:** Lifestyle characteristics of the AMBER cohort at baseline (*N* = 1,528) for the Calgary (*n* = 884) and Edmonton (*n* = 644) sites

Lifestyle characteristic	Total cohort		Calgary		Edmonton	
	*N* = 1528		*n* = 884		*n* = 644	
	Mean	SD	Mean	SD	Mean	SD
Smoking status						
Never smoker, n, %	867	56.7	488	55.2	379	58.9
Ever smoker, n, %	563	36.8	333	37.7	230	35.7
Current smoker, n, %	98	6.4	63	7.1	35	5.4
Pack-years	11.2	11.2	11.3	1117	11.1	13.3
Alcohol consumption (grams of ethanol/day)	7.1	16.2	7.5	17.6	6.6	14.2
Diet and macronutrient intake						
Daily caloric intake (kilocalories/day)	1718	747	1662	693	1791	806
Protein (grams/day)	72	33	69	32	75	34
Fat (grams/day)	68	35	66	33	70	36
Carbohydrates (grams/day)	203	95	195	84	214	107

*SD* standard deviation

The comprehensive assessment of health-related fitness at baseline is provided in Table 4. AMBER participants had an average weight of 73.8 kg (SD = 15.9), height of 163.8 cm (SD = 6.5), and a BMI of 27.5 (SD = 5.6). Only 37.8% of the cohort had a BMI within the normal range, with the majority having overweight (34.3%) or obesity (27.9%). Their average waist-to-hip ratio was 0.88 with an average waist circumference of 92.9 cm (SD = 13.5) and hip circumference of 105.9 (SD = 11.6). From the DXA scan, their percent body fat was 41.6% (SD = 7.3) and lean body mass was 54.2% (SD = 8.3).

**Table 4:** Health-related fitness measures of the AMBER cohort at baseline (*N* = 1,528) for the Calgary (*n* = 884) and Edmonton (*n* = 644) sites

Health-related fitness measure	Total cohort		Calgary		Edmonton	
	*N* = 1528		*n* = 884		*n* = 644	
	Mean	SD	Mean	SD	Mean	SD
Body composition						
Weight (kg)	73.8	15.9	73.7	15.7	74.0	16.1
Height (cm)	163.8	6.5	163.6	6.6	164	6.3
Body mass index (kg/m^2^)	27.5	5.6	27.5	5.5	27.5	5.8
Normal, n, %	578	37.8	328	37.1	250	38.8
Overweight, n, %	524	34.3	303	34.3	221	34.3
Obese, n, %	426	27.9	253	28.6	173	26.9
Waist circumference (cm)	92.9	13.5	94.2	13.2	91.1	13.8
Hip circumference (cm)	105.9	11.6	106	11.6	105.8	11.7
Waist-to-hip ratio	0.88	0.07	0.89	0.06	0.86	0.07
Body fat (%)	43.1	7.1	43.5	6.7	42.5	7.6
Body fat mass (kg)	31.8	11.5	32.0	11.0	31.5	12.1
Lean body mass (%)	53.7	6.6	53.4	6.3	54.1	7.1
Lean body mass (kg)	37.8	5.4	37.6	5.6	38.1	5.2
Fat-free mass (kg)	41.6	4.5	39.7	4.5	44.2	4.6
Lean body mass/fat free mass ratio	0.9	0.0	0.9	0.0	0.9	0.0
Physical fitness measurements						
Sit and reach (cm)	27.4	9.9	26.6	10.1	28.6	9.6
Partial curl-ups (#)	29	29	26	28	32	32
Combined grip strength (kg)	55	12	57	13	53	11
VO_2peak_						
Absolute (L/min)	1.89	0.37	1.87	0.37	1.91	0.37
Relative (ml/kg/min)	26.6	6	26.4	6.1	26.9	5.8
Chest press strength (kg)	27.9	7.9	29.3	7.8	26.2	7.6
Chest press endurance (repetitions)	27	9	26	10	28	9
Leg press strength (kg)	74.6	24.4	73.2	23	76.6	26.2
Leg press endurance (repetitions)	19	10	17	9	21	11
Upper body function						
Presence of lymphedema, n, %^a^	53	3.5	30	3.5	23	3.6
Range of motion limitation^b^						
Shoulder abduction, active, n, %	487	32.1	319	36.5	168	26.2
Shoulder flexion, active, n, %	330	21.8	203	23.3	127	19.8
Shoulder horizontal abduction, active, n, %	193	12.9	146	17.1	47	7.4

*SD* standard deviation, *kg* kilograms, *cm* centimeters, *VO*_*2*_ volume of oxygen consumed per minute

^a^ ≥ 200 ml volume difference between affected and unaffected arms

^b^ > 10° difference in range of motion between affected and unaffected arm

Variables with > 5% of total cohort with missing data: partial curl-ups (9%), VO_2_ peak (24%), chest press (16%), and leg press (17%)

AMBER participants’ average cardiopulmonary fitness (VO_2peak_) was 26.6 ml/kg/minute (SD = 6). Their flexibility, as assessed with the sit and reach test, was 27.4 cm (SD = 9.9). They completed an average of 29 (SD = 29) curl-ups on the test for abdominal endurance. Their combined right and left grip strength was 55 kg (SD = 12) and upper body strength as indicated by the chest press predicted 1-RM was 27.9 kg (SD = 7.9). Their lower body strength assessed by the leg press predicted 1-RM was 74.6 kg (SD = 24.4).

At baseline, 3.5% of the AMBER study population had lymphedema. There was also evidence of limited range of motion for shoulder abduction (32.1%), flexion (21.8%), and horizontal abduction (12.9%).

Physical activity and sedentary behavior measures from both the self-report and accelerometry-derived measures are presented in Table 5. The AMBER participants reported doing 18.6 MET-hours/day of total physical activity in the year before their diagnosis that was composed of mainly household (8.6 MET-hours/day) and occupational (5.9 MET-hours/day) activity, with less recreational activity (4.1 MET-hours/day). The total self-reported sitting time/day was 9.4 h. Device-based assessment indicated participants spent most of their time being sitting, as estimated from the activPAL® device, (8.9 h/day, SD = 1.7). The Actigraph® indicated participants engaged in 4.4 h/day (SD = 1.2) of light-intensity activity, 0.9 h (SD = 0.5) of moderate-intensity activity, and 0.2 h (SD = 0.2) of vigorous-intensity activity. They achieved an average of 7,348 steps/day (SD = 3109). Both devices were worn for approximately 14 h/day on average and there were at least 5.5 valid days per week of wear time.

**Table 5:** Self-report and accelerometry-derived measures of physical activity and sedentary behavior of the AMBER cohort at baseline (*N* = 1,528) for the Calgary (*n* = 884) and Edmonton (*n* = 644) sites

Physical activity measure	Total cohort		Calgary		Edmonton	
	*N* = 1528		*n* = 884		*n* = 644	
	Mean	SD	Mean	SD	Mean	SD
Self-reported past-year physical activity						
Total non-sedentary physical activity						
MET-hours/day	18.6	9.1	17.9	8.9	19.5	9.2
Hours/day	5.8	2.6	5.6	2.6	6.1	2.7
Occupational activity						
MET-hours/day	5.9	6.4	5.6	6.3	6.3	6.5
Hours/day	1.8	1.8	1.7	1.8	1.9	1.8
Household activity						
MET-hours/day	8.6	5.8	8.2	5.8	9.1	5.8
Hours/day	3.1	1.9	3.0	1.9	3.3	1.9
Recreational activity						
MET-hours/day	4.1	3.8	4.1	3.8	4.1	3.9
Hours/day	0.9	0.8	0.9	0.8	0.9	0.8
Self-reported sedentary time (typical week)						
Total sitting (hours/day)	9.4	3.5	9.3	3.4	9.3	3.8
Recreational sitting (hours/day)	6.0	2.8	6.0	2.8	6.0	2.8
Occupational sitting (hours/day)	2.0	2.3	2.0	2.3	2.0	2.3
Travel sitting (hours/day)	1.3	1.0	1.3	1.0	1.4	1.2
Accelerometry-derived measures Actigraph® from Soj3x^a^						
Total wear time (hours/day)	14.0	1.3	14.0	1.3	13.9	1.3
Total valid wear time (days)	5.5	1.4	5.3	1.2	5.7	1.6
Sedentary time (hours/day)	8.5	1.5	8.5	1.5	8.5	1.5
Light activity (hours/day)	4.4	1.2	4.4	1.2	4.4	1.3
Moderate activity (hours/day)	0.9	0.5	0.9	0.5	0.8	0.5
Vigorous activity (hours/day)	0.2	0.2	0.2	0.2	0.2	0.2
Actigraph® from vertical axis*						
Sedentary time (hours/day)	8.7	1.5	8.8	1.5	8.6	1.4
Light activity (hours/day)	3.7	0.9	3.7	0.9	3.7	0.9
Lifestyle MVPA (hours/day)	1.2	0.5	1.2	0.6	1.2	0.5
Ambulatory MVPA (hours/day)	0.4	0.3	0.4	0.4	0.4	0.3
activPAL®						
Total wear time (hours/day)	14.2	1.2	14.2	1.3	14.2	1.2
Total valid wear time (days)	5.9	1.5	5.8	1.5	5.9	1.5
Sedentary time (hours/day)	8.9	1.7	9.0	1.7	8.9	1.6
Total steps (steps/day)	7348	3109	7368	3176	7322	3021
Standing time (hours/day)	3.8	1.3	3.7	1.3	3.8	1.3
Stepping time (hours/day)	1.5	0.6	1.5	0.6	1.5	0.6

*SD* standard deviation, *MET* metabolic equivalent of task, *MVPA* moderate-to-vigorous physical activities

^a^Total Actigraph*®* wear time and valid wear time are identical between two methods: Soj3x and vertical axis counts/minute (cpm); the cut-off points for vertical axis are sedentary as < 100 cpm, light activity as 100–760 cpm, lifestyle MVPA as ≥ 760 cpm, and Ambulatory MVPA as ≥ 2020 cpm

## Discussion

The AMBER Study is a unique breast cancer survivor cohort with 1,528 women recruited between 2012 and 2019. Participants have provided detailed and comprehensive self-report and direct measurements of a wide range of exposures shortly after diagnosis (baseline) and again at 1-, 3-, and 5-year follow-up. These participants will be followed for at least an additional five years for all cancer survival outcomes.

Initial recruitment into the cohort required seven years to achieve the target sample size of 1,500 women. The final sample is 10% of all Albertan women who were diagnosed with breast cancer during this period. The eligibility requirements for this cohort study reduced the available sample size for recruitment to 3,673 breast cancer patients (25%) of which 42% were recruited into the AMBER cohort. This recruitment rate is similar to comparable breast cancer survivor cohort studies, including the Pathways Study [37], Health, Eating, Activity, and Lifestyle (HEAL) Study [38], and Life After Cancer Epidemiology (LACE) Study [39], that have recruited between 37% and 41% of eligible breast cancer survivors. Since our study required time and effort by participants to complete comprehensive measures of physical health at multiple time points post-diagnosis, the recruitment rate is notable. Specifically, AMBER participants, unlike participants in previous cohort studies, had to complete two days of health-related fitness testing in addition to providing blood draws, completing several questionnaires, and wearing two monitoring devices for one week at multiple time points. The main reasons for study refusal were being overwhelmed/too sick or living out of town and having too far to travel for the testing. We mitigated these issues as much as possible by accommodating single-day visits and adjusting the fitness tests, when necessary, to include as many women as possible.

For those women who did participate, we had high baseline completion rates for all the assessments. In AMBER, we achieved 99.9% baseline completion for anthropometric measurements which is comparable to the 84%–100% rate reported in other breast cancer cohorts [37–39]. We have DXA measures on 98.3% of our participants, a gold standard measure of body composition that has not been included in previous studies. We also collected baseline blood samples from 98.9% of participants. To our knowledge, the Pathways Study [37] is the only other cohort to collect baseline blood samples and reported a completion rate of 86%.

We compared our population to the base population of breast cancer survivors in Alberta and the USA, as well as to other breast cancer survivor cohorts in the USA to assess the generalizability of the AMBER cohort study. The most recent publication of the age and stage distribution of women diagnosed with stage I to III breast cancer in Alberta between 2016 and 2017 [40] reported 48% stage I, 36% stage II, and 10% stage III cases at diagnosis compared to the AMBER distribution of 45% stage I, 47% stage II, and 9% stage III. The small differences between the AMBER sample stage distributions compared with the entire Alberta population can be attributed to the exclusion of cases under T1c. The stage distribution is comparable to the American Cancer Society, Centers for Disease Control and Prevention, the National Cancer Institute, and the North American Association of Central Cancer Registries joint 2018 annual report in which the stage distribution for female breast cancer was 46.5% stage I, 31.6% stage II, and 10.8% stage III from 1999 to 2015 [41]. The stage distribution of AMBER study participants is comparable to other breast cancer survivor cohorts in the USA [37–39]. Since we excluded lower stage I breast cancers (i.e., below T1c), we anticipate that the overall and breast cancer-specific event rates in AMBER will exceed the event rates in other established lifestyle and breast cancer survivor cohorts. The rates of ER positive (88%), PR positive (77%), and HER-2 positive (16%) tumors in the AMBER cohort were higher than in the Pathways Study (82%, 69%, and 10%, respectively) [37].

AMBER participants, who were on average 55 years at diagnosis, were somewhat younger than the base population of female breast cancer survivors in Alberta [40] as well as the participants in the Pathways [37] and LACE [39] studies who were 60 years at diagnosis; and in the HEAL cohort who were, on average, 58 years at diagnosis [38]. The main reason for our younger sample is likely the requirement for higher-level functioning for maximal fitness testing which may have introduced an age bias. Our participants were also mostly White, married, well educated, and had higher household incomes which make them somewhat less representative of the entire breast cancer population in Alberta. In addition, besides their cancer diagnosis, the AMBER participants had few co-morbidities at the time of diagnosis (on average 0.5 conditions/participant) making this cohort relatively healthier than participants in the LACE study which provided a detailed description of co-morbid conditions of their cohort at baseline [39].

The rate of 6% for current smokers in AMBER was comparable to the rates reported in other breast cancer cohorts (7%–9%) [38, 39] but were lower than the Canadian population rate of 12.3% for females [42]. AMBER participants consumed, on average, one-half alcoholic drink per day which aligns with recommendations for alcohol drinking in women for cancer prevention in Canada [43]. Their daily caloric intakes were lower than expected for their average body weights which might be attributed to the under-reporting of dietary intake that occurs with food frequency questionnaires [44]. AMBER study participants had an average BMI of 27.5 kg/m^2^ which classified them as overweight and 27.9% of the cohort had obesity (BMI ≥ 30) at baseline. Their average body fat percentage of 43.1% was above the recommended range for women between the ages of 35 and 55 years (23%–38%) and for women over the age of 56 years (25%–38%) [45]. Participants’ average waist circumferences of 92.9 cm also exceeded the recommended healthy level of 88 cm [46].

With respect to health-related fitness at baseline, the average cardiorespiratory fitness level (VO_2peak_) of AMBER participants was borderline fair according to age- and sex-specific norms for maximal and submaximal exercise tests [47]. The mean score of 55 kg for combined grip strength would classify participants as *very good*, the mean score of 25 for curl-ups would classify participants’ abdominal muscular endurance as *excellent* and the mean score of 27.4 cm for the sit and reach test would classify participants’ low back and hip flexibility as *fair* according to age- and sex-specific norms [47, 48]. A low percentage (3.5%) of AMBER participants experienced lymphedema at baseline and approximately one-third of the population experienced some range of motion limitations. We expect that the number of participants presenting lymphedema will increase between baseline and Year 1 follow-up. However, it is also possible that cancer-related lymphedema was underestimated at baseline since a calculated volume differential of ≥ 200 ml between arms can be used to rule in lymphedema, but volumes < 200 ml cannot be used to rule out lymphedema [30].

Although AMBER participants’ self-reports and direct measures of physical activity appear to exceed the recommended levels of 150–300 min/week of moderate-to-vigorous-intensity physical activity [49], their values are comparable to other studies using similar measurement methods [50, 51]. Based on the accelerometer data, participants were achieving 0.9 h/day of moderate activity and 0.2 h/day of vigorous activity. These participants were also spending 8.9 h/day, or 63% of the waking day, being sedentary as recorded by the activPAL®, which is consistent with results for middle aged and older US women [50, 52]. From the self-reported physical activity data, it was evident that most of their non-sedentary time (84%) was spent in occupational and household activity which is typical for women of this age group.

With the successful completion of the baseline assessments, we have established a cohort of breast cancer survivors with detailed and comprehensive measures that will provide novel insights into the roles of physical activity, sedentary behavior, and health-related fitness in optimizing breast cancer survivorship. These insights will be translated into evidence-based targeted guidelines on these topics, including a precision medicine approach. Additional study strengths include the prospective design, timing of recruitment (soon after diagnosis), the exclusion of lower stage (< T1c) breast cancer, the standardized and direct measurements of outcomes that are repeated at three time points during follow-up, and our high measurement completion rates. While we have achieved excellent internal validity of the cohort, there are some limits to the external generalizability of the sample given the relatively homogeneous demographic, medical, and behavioral profiles of study participants. Initial publications from our cohort study will describe the baseline patient-reported outcomes and their associations, the physical activity, sedentary behavior, and health-related fitness measures. The cohort follow-up is planned to continue for at least another eight years to describe the associations of these measures with long-term outcomes, including recurrence and survival.

## Supplementary Information

Below is the link to the electronic supplementary material.

Supplementary file1 (docx 14 KB)

## Data Availability

The data that support the findings of this study are available from the corresponding author upon reasonable request.

## Code availability

The SAS® and STATA® codes used for these analyses are available upon request.
