# Supplementary material for: The Alberta moving beyond breast cancer (AMBER) cohort study: baseline description of the full cohort
Source: Cancer Causes Control. 2022 Jan 22;33(3):441–53. doi: 10.1007/s10552-021-01539-6 (PMC8821077; doi:10.1007/s10552-021-01539-6)
Supplement: Supplementary file 1 — Supplementary file1 (docx 14 KB) [file 10552_2021_1539_MOESM1_ESM.docx]

**Supplementary Table 1.** Assessment completion rates for baseline participants in the AMBER Cohort Study, Alberta, 2012-2019

| **Assessment** | **Calgary**  **(n=884)**  **n (%)** | **Edmonton**  **(n=644)**  **n (%)** | **Total**  **(n=1528)**  **n (%)** |
| --- | --- | --- | --- |
| **Health-related fitness** | |  |  |
| Treadmill tests attempted | 799 (90.4%) | 589 (91.5%) | 1388 (90.8%) |
| VO_2peak_ | 652 (73.8%) | 512 (79.5%) | 1164 (76.2%) |
| Estimated VO_2max_^a^ | 73 (8.3%) | 50 (7.8%) | 123 (8.0%) |
| No VO_2_ data | 74 (8.4%) | 27 (4.2%) | 101 (6.6%) |
| Upper body strength | 704 (79.6%) | 580 (90.1%) | 1284 (84.0%) |
| Upper body endurance | 691 (78.2%) | 579 (89.9%) | 1270 (83.1%) |
| Lower body strength | 762 (86.2%) | 576 (89.4%) | 1338 (87.6%) |
| Lower body endurance | 741 (83.8%) | 571 (88.7%) | 1312 (85.9%) |
| Grip strength | 848 (95.9%) | 621 (96.4%) | 1469 (96.1%) |
| Curl-ups | 798 (90.3%) | 597 (92.7%) | 1395 (91.3%) |
| Flexibility | 830 (93.9%) | 618 (96.0%) | 1448 (94.8%) |
| Anthropometric measurements | 883 (99.9%) | 643 (99.8%) | 1526 (99.9%) |
| DXA scan | 866 (98.0%) | 636 (98.8%) | 1502 (98.3%) |
| **Clinical data** |  |  |  |
| Lymphedema | 867 (98.1%) | 642 (99.7%) | 1509 (98.8%) |
| Upper arm range of motion | 878 (99.3%) | 642 (99.7%) | 1520 (99.5%) |
| Balance | 865 (97.9%) | 642 (99.7%) | 1507 (98.6%) |
| Blood samples | 879 (99.4%) | 632 (98.1%) | 1511 (98.9%) |
| **Questionnaires** |  |  |  |
| Baseline Health Questionnaire | 884 (100%) | 644 (100%) | 1528 (100%) |
| Diet History Questionnaire | 825 (93.3%) | 625 (97.0) | 1450 (94.9%) |
| General Health Questionnaire | 832 (94.1%) | 627 (97.4%) | 1459 (95.5%) |
| Past Year Physical Activity Questionnaire | 831 (94.0%) | 626 (97.2%) | 1457 (95.4%) |
| **Activity monitors** |  |  |  |
| ActiGraph® | 833 (94.2%) | 624 (96.9%) | 1457 (95.4%) |
| activPAL® | 823 (93.1%) | 618 (96.0%) | 1441 (94.3%) |

^a^Estimated from submaximal direct measures of VO_2._

Notes: DXA=dual energy x-ray absorptiometry scans.
